# Role of nurses in medication management at the end of life: a qualitative interview study

**DOI:** 10.1186/s12904-020-00574-5

**Published:** 2020-05-13

**Authors:** Bregje A. A. Huisman, Eric C. T. Geijteman, Marianne K. Dees, Noralie N. Schonewille, Margriet Wieles, Lia van Zuylen, Karolina M. Szadek, Agnes van der Heide

**Affiliations:** 1 Department of Anesthesiology, Amsterdam UMC, Vrije Universiteit Amsterdam, De Boelelaan 1117, 1081 HV Amsterdam, Netherlands; 2 Hospice Kuria, Amsterdam, Netherlands; 3 Department of Medical Oncology, Erasmus MC Cancer Institute, Rotterdam, Netherlands; 4 Department of Public Health, Erasmus University Medical Center, Rotterdam, Netherlands; 5 Department of Primary and Community Care, Radboud University Medical Center, Nijmegen, Netherlands; 6 Department of Gynaecology, OLVG West, Amsterdam, the Netherlands

**Keywords:** Nursing, Palliative care, End-of-life care, Polypharmacy, Drug therapy, Medication therapy management, Interdisciplinary communication, Decision making, Interview

## Abstract

**Background:**

Patients in the last phase of their lives often use many medications. Physicians tend to lack awareness that reviewing the usefulness of medication at the end of patients’ lives is important. The aim of this study is to gain insight into the perspectives of patients, informal caregivers, nurses and physicians on the role of nurses in medication management at the end of life.

**Methods:**

Semi-structured interviews were conducted with patients in the last phase of their lives, in hospitals, hospices and at home; and with their informal caregivers, nurses and physicians. Data were qualitatively analyzed using the constant comparative method.

**Results:**

Seventy-six interviews were conducted, with 17 patients, 12 informal caregivers, 15 nurses, 20 (trainee) medical specialists and 12 family physicians. Participants agreed that the role of the nurse in medication management includes: 1) informing, 2) supporting, 3) representing and 4) involving the patient, their informal caregivers and physicians in medication management. Nurses have a particular role in continuity of care and proximity to the patient. They are expected to contribute to a multidimensional assessment and approach, which is important for promoting patients’ interest in medication management at the end of life.

**Conclusions:**

We found that nurses can and should play an important role in medication management at the end of life by informing, supporting, representing and involving all relevant parties. Physicians should appreciate nurses’ input to optimize medication management in patients at the end of life. Health care professionals should recognize the role the nurses can have in promoting patients’ interest in medication management at the end of life. Nurses should be reinforced by education and training to take up this role.

## Background

Medication management is one of the components of palliative care that aims to improve the quality of life of patients facing a life-limiting disease. Medication management in palliative care is challenging. Despite all resources, patients’ symptom load is generally high [1–4]. The broad scope of palliative care, encompassing different illnesses and recently diagnosed incurable disease as well as the terminal phase, makes it even more complex. Disadvantages of some medications may outweigh the benefits at the end of life. Potentially inappropriate medications (PIMs) are medications that should be reconsidered because they have a significant risk of adverse events, for which there is evidence for an equally or more effective but lower-risk alternative therapy, medications that are prescribed at a higher frequency or for a longer period than clinically indicated, or medications with recognized drug–drug or drug–disease interactions [5]. This applies particularly to medications prescribed for the treatment of comorbidities or long-term prevention [6–9]. In practice, timely withdrawal of potentially inappropriate medications (PIMs) is not self-evident. As a result, many PIMs are continued until the very end of life [10, 11].

Physicians are primarily responsible for pharmacological treatment [12]. However, several studies show that it is not self-evident that physicians review medication used by patients with a limited life expectancy and that they report a variety of reasons for not considering deprescribing of PIMs. Perhaps the most important reason for not considering deprescribing is limited awareness [13]. Other reasons are it is given low priority, uncertainty about the consequences of deprescribing medications, and avoiding confronting patients with their impending death [13, 14]. Moreover, barriers to adequate medication management exist on the level of the organization of health care, namely the lack of organizational support, shortage of staff, lack of time and difficult-to-access patient medical records [15, 16]. Other barriers occur in communication and collaboration between physicians and nurses [15], particularly in the interfaces between primary, secondary and tertiary care. Further, the nature of the relationship between physician and nurse and the hierarchies in place may hinder nurses from actively discussing their suggestions regarding medication [15].

The barriers on the physicians’ side and the interdisciplinarity of end-of-life care, raise questions about the role of nurses in medication management. Nurses have an important role in palliative care and alleviation of symptoms [17, 18]. A qualitative systematic meta-synthesis showed nurses’ views on their role in providing palliative care. They feel they contribute to the coordination and continuity of care and to ensuring that care is patient-centred and adequately addresses patients’ needs [19]. Literature about the role of nurses in medication management in palliative care is scarce. Some studies describe the considerable role of nurses in addressing polypharmacy in older people at home or in long-term care facilities [20–22]. Others highlight their role in the administration of palliative chemotherapy [23] or administration of anticipatory medications for people who are dying [24–26]. Wilson et al. found that there are significant differences in the level of education of nurses, specialized nurses and nurse practitioners that could affect their role in medication management [25]. Previous studies about the role of nurses in palliative care or medication management are mainly based on the perspective of nurses themselves [19, 23–25, 27]. We studied the perspectives of patients, informal caregivers, nurses and physicians on the role of nurses in medication management at the end of life.

## Methods

### Design

This qualitative interview study is part of the MEDIcation management in the LAST phase of life (MEDILAST) research project [11, 13, 16, 28]. The purpose of the primary study was to gain insight into the norms and values of patients, informal caregivers and health care providers regarding medication management at the end of life, defined as the last 3 months of life [16]. We performed a secondary analysis of their perspectives on the role of nurses in medication management at the end of life.

### Participants

We asked physicians working in academic and general hospitals (geriatrics, neurology, oncology, cardiology and pulmonology departments), hospices and general practices, whether they cared for a patient who fulfilled the following inclusion criteria: 1) an estimated life expectancy of less than 3 months; 2) patient knew his/her life expectancy was limited; 3) and was able to participate in an interview. When eligible, the physician would inform the patient about the study and ask them whether the researchers could approach them for an interview. Only one patient per physician was included.

Patients received written information about the study from the researchers and were asked to sign an informed consent form prior to the interview. Afterwards, the patient was asked for permission to ask an informal caregiver, nurse, family physician and clinical specialist involved in the patient’s care to participate in an interview. Purposive sampling was used to ensure diversity of patients’ age, diagnosis, social background, and religious beliefs. All participants were requested to complete a short questionnaire regarding their background. The questionnaire for the nurses and physicians also included questions regarding their professional career, their role in care for the patient, and their views on medication management. In the Netherlands nurses do not have a (de) prescriptive authority, unlike nurse specialists who formally can (de) prescribe medication. We only included nurses in our study, no nurse specialists [16].

### Data collection

Data for this study were collected from October 2013 to February 2015. Face to face, semi-structured interviews were conducted by the first, second and third author, Jimmy Arevalo (J.A.) and Roberto Perez (R.P.). The interviewers included an anesthesiologist (MD), an internal medicine resident-researcher (MD), a general practitioner (MD, PhD), an anesthesiology resident-researcher (MD) and a senior researcher in pain and palliative care (PhD), with experience in qualitative research. We used a topic list (see Table 1) that was developed by the project team, guided by a review of the literature on medication management and consultation of ten specialists in palliative care, including a nurse, physicians and pharmacologists [29]. The topic list was refined based on new insights during the interviews. The interviews lasted 30–60 min [16].

**Table 1:** Topic list

**General**	
• Thoughts and opinions about medication use	
• Medication decision-making	
• Preventive and chronic medication in the last phase of life	
• Medication deprescription	
• Communication regarding medication	
• Who were involved; their responsibilities and roles	
**Role of the nurse**	
• What is your opinion about the role of the nurse in decision-making about medication?	
• In your opinion, which role can the nurse have in decision-making about medication?	
• What is your experience regarding the collaboration of your physician and nurse relating to decision-making on the use of medication?	
• Does the nurse have enough knowledge about the use of medication?	
• Suppose the nurse would suggest your physician to … adjust/start/stop … what do you think about that?	
• Suppose you/the patient would be at home/in hospital/in hospice? Who would be in charge of decision-making with respect to medication? What is the role of the nurse?	

### Data analysis

The interviews were audio-recorded and transcribed verbatim. In contrast to the primary study in which the transcripts were analyzed in order of collection, for this secondary analysis we randomly analyzed transcripts per patient case, using the constant comparative method. This qualitative method is part of the grounded theory approach in which concepts emerge as theory is formed [30]. The analysis was performed with the aid of qualitative research software (ATLAS.ti version 7.5.6). Two researchers (first and fourth author) independently read through the interviews and attached open codes to all issues that seemed relevant to provide insight into the role of the nurses. The analysis was performed in five rounds of 13–15 interviews each. The coders met after each round to discuss the evolving code list, deduce possible themes and adjust the code book if needed. This deductive process, the interpretation of codes and discrepancies were regularly discussed with the team members (second author and R.P.). The final codes, themes and categories were discussed with the whole project group.

## Results

In total 76 interviews were conducted, including 17 patients (one included patient could not be interviewed due to her condition), 12 informal caregivers, 15 nurses, 20 (trainee) medical specialists and 12 family physicians. Characteristics of the participants are presented in Table 2. The nurses were between 23 and 59 years old (mean 47.1 years), 80% were female, and they had a working experience in nursing of 3–38 years (mean 20.8 years). The majority of the nurses received a specialty education, in palliative care [4], oncology [4], geriatrics [2], dialysis [1] or occupational therapy [1].

**Table 2:** Participants’ characteristics

Case number	Patient characteristics						Informal caregiver characteristics	Nurse characteristics			Medical specialist characteristics	Family physician characteristics
	Gender, age (years)^a^	Diagnosis	Survival after interview (days)	Marital status	Religion	Country of origin	Relation, age (years)^a^	Gender, age (years)^a^	Specialty	Setting	Gender, age (years)^a^, specialisation	Gender, age (years)^a^
1	M 80^+^	Kidney failure	30	Partnership	–	Netherlands	Wife 70^+^	M 50^+^	Dialysis	General hospital	M 30^+^ internist	M 60^+^
2	F 60^+^	Melanoma	35	Married	Protestant	Netherlands	Husband 60^+^	F 30^+^	Oncology	Academic hospital	F 30^+^ internist	M 50^+^
3	F 60^+^	COPD	12	Single	Catholic	Germany	–	F 20^+^	Oncology	General hospital	M 50^+^ pulmonologist	F 20^+^
4	F 80^+^	Dementia	8**	Widow	Islamic	Turkey	Grandson 20^+^	F 20^+^	Oncology	Academic hospital	F 30^+^ trainee geriatrician F 30^+^ internist	M 30^+^
5	F 60^+^	Stomach cancer	***	Divorced	Catholic	Suriname	–	–	–	–	M 60^+^ oncologist	–
6	M 70^+^	Acute myeloid leukaemia	67	Married	–	Netherlands	–	M 50^+^	Oncology	Academic hospital	F 50^+^ haematologist F 30^+^ trainee haematologist	–
7	M 50^+^	Lung cancer	35	Single	Buddhist	Netherlands	Sister 50^+^	F 50^+^	Geriatrics, palliative care	Hospice	–	M 30^+^
8	F 80^+^	Colon cancer	5	Widow	Catholic	Netherlands	–	M 50^+^	Palliative care	Hospice	F 40^+^ oncologist	–
9	M 60^+^	Mesothe- lioma	54	Married	–	Netherlands	Wife 50^+^	F 50^+^	–	Hospice	M 30^+^ pulmonologist M 60^+^ anaesthesiologist	–
10	M 70^+^	Esophagus cancer	19	Widower	–	Netherlands	Daughter-in-law 40^+^	F 40^+^	–	Hospice	F 40^+^ elderly care	F 30^+^
11	F 50^+^	Lung cancer	63	Divorced	Protestant	Netherlands	Sister 40^+^	F 50^+^	Palliative care	Hospice	F 50^+^ elderly care M 40^+^ trainee elderly care F 30^+^ pulmonologist	–
12	M 80^+^	Stomach cancer	117	Widower	Reformed	Netherlands	Son 40^+^	F 50^+^	Palliative care	Hospice	F 30^+^ trainee elderly care	–
13	M 60^+^	Esophagus cancer	54	Married	–	Netherlands	–	–	–	–	F 40^+^ oncologist	M 30^+^
14	M 90^+^	Cardiac failure	96	Widower	–	Germany	Daughter 60^+^	F 50^+^	Geriatrics	Nursing home	M 20^+^ trainee cardiology	F 40^+^
15	F 70^+^	Mouth cancer	43	Married	–	Netherlands	Female friend 70^+^	F 40^+^	–	Home care	–	M 50^+^
16	F 60^+^	Lung cancer	31	Married	Catholic	Netherlands	–	–	–	–	F 30^+^ trainee pulmonologist	M 40^+^
17	F 40^+^	ALS	***	Married	Catholic	France	Son#	F 50^+^	#	Home care	M 30^+^ rehabilitation specialist	F 30^+^ trainee
18	M 80^+^	Old age, cardiac failure, COPD	***	Married	Catholic	Netherlands	Daughter 50^+^	F 40^+^	Occupational therapy	Home care	–	M 40^+^

*M* Male, *F* Female

*ALS* Amyotrophic lateral sclerosis, *COPD* Chronic obstructive pulmonary disease

^a^ Age given in ranges: 20^+^: 20–29 years; 30^+^: 30–39 years etc.

**Could not be interviewed

***Survival longer than 6 months

# Missing

A minority of the nurses reported having discussed discontinuation of medication with the patient. They reported to be involved with: activities of daily living, administration of medication, wound care, tube feeding, nursing technical procedures, psychological care, social care, care for family, information, instruction and education, supportive conversations, kidney replacement therapy, supervising blood transfusion or chemotherapy, treatment plan monitoring, coordination of care and/or nursing and palliative care in general.

Patients, informal caregivers, nurses and physicians seemed to agree that nurses can act as a linchpin [19] in medication management at the end of life. The roles of the nurse could be categorized into roles towards the patient, towards the informal caregiver and towards the physician (see Table 3).

**Table 3:** Categories and codes regarding the role of the nurse in medication management at the end of life

Relation	Category (role)	Codes
Nurse - patient	Inform	Instruction intake medication, (side) effects of medication, adjustments of medication, how to monitor effect, dosage form, explanation of necessity of medication
	Signal	Intake: Problems with intake or route of administration, adherence, aversion and motivation, medication alternatives, polypharmacy Side effects: Interactions, influence on quality of life, monitoring (e.g. defecation), diagnose overdose, common side effects, effect of reduction of medication Clinical assessment: Clinical changes, appearance of symptoms, comfort centrally, pain perception, trigger for changes in treatment plan, effect of medication, symptom assessment tools Multidimensional vision: Attention for underlying emotions of the patient, importance of medication to the patient, influence of cognitive status, psychological wellbeing, social dimension (relations, environment), contribution to personalized pharmacological therapy, take into account character of the patient
	Represent	Patient discusses wishes regarding medication with or via nurse, nurse is first contact person, nurse lends an ear to patient, nurse expresses to physician how patient is doing, nurse receives more information from patient than physician, attention on the patient, nurse records in file; which role patient wants to have in medication management, motivate, put wishes and values of the patient at the centre, explore aversion towards medication, assist in decision-making and mourning, guard over the autonomy of the patient, support in quality of life, confidential advisor
	Support	Activities of Daily Living: order, obtain and prepare medication
		Intake: self-administration of ‘as needed’ medication, facilitate intake of medication
		Feasibility: Propose change of route of administration, financial aspects, consultation of pharmacist as needed
		Complementary care: Non-pharmacological supportive interventions
Nurse - informal caregiver	Inform	Instruction intake, (side) effects of medication, adjustments of plan, how to monitor effect, dosage form, necessity of medication
	Support	Evaluate informal caregiver capacity, contact person for questions
	Involve	Involve informal caregiver in case patient cannot express wishes or complaints, link between physician and informal caregiver, asses which role informal caregiver can have in medication management, informal caregiver can substitute for nurse
Nurse - physician	Inform	Report observations regarding effects, side-effects, clinical assessment and other issues to physician
	Support	Give input at multidisciplinary meeting/grand round/periodical meeting, think along with physician about inappropriate or lacking medication, control function, team decision-making in medication management, physician and nurse complement each other, final decision regarding medication by physician, no role for nurse in decision-making Initiative: Proposal of adjustment of medication to physician, make medication discontinuation a subject of discussion, as needed medication for symptom control, prevention of symptoms, advance care planning, support reduction of medication, check for contraindications
	Represent	Repeat information from physician to patient Continuity: Daily contact with patient, more frequent and prolonged contact than with physician, periodical evaluation, good insight into the course of the disease, 24-h availability

### Nurse-patient

Patients, informal caregivers, nurses and physicians stated that, in medication management at the end of life, nurses have four important roles in relation to patients: to inform, signal, represent and support. They report that nurses give instructions for the intake of medication, inform about the possible (side) effects of medication and about adjustments of the treatment plan. Nurses also inform patients about how the effect of adjustments in medication will be monitored, about dosage forms and the necessity of medication.

Participants also mentioned how nurses play a role in the clinical assessment related to medication use. They stated that nurses have a role in the assessment of the burden of medication intake.

*“Observing, reporting, … but [nurses] also monitor the intake of medication, do the patients take them? If they don’t take them, register this well. If they regularly resist to take them or cannot swallow, then I should know this and have a conversation with the patient about this.”* (Medical specialist 10)

A specific aspect that only the nurses themselves highlighted, is the importance of a multidimensional assessment and approach, taking into account physical, psychological, social and spiritual issues.

*“Interestingly, I never realized when I started working there, that the physicians don’t really know the patient. They know he needs help with washing and his pills, but what someone did in his life or what’s important to him, they don’t know. … Well they like to hear the other side of the story, and say ‘interesting life story’, that you actually need to know to be able to deliver good care. You should know what is important to someone, if he grieves about things that happened in life. How he copes with health-related skills, medication adherence, health-promoting behavior, if that never interested him … that’s his autonomy.”* (Nurse 14)

Moreover, nurses specifically pay attention to possible side effects of the medications, their interactions and their impact on quality of life.

*“[The nurse] observes him and sees him more than we do, so indeed she can monitor how much pain he has in the night and how it goes with washing. So I think the nurse is a good indicator to determine whether he needs more medication.”* (Informal caregiver 10)

Participants also described the role of the nurse as being a representative for the patient. Patients discuss wishes regarding medication with or via the nurse and the nurse informs the physician how the patient is doing. Nurses receive more information from patients than the physician. They are considered the first contact person, who lends an ear to the patient and closely watches the patient.

Lastly, participants described how the nurses support the patient with practical aspects concerning their care and medication use.

*“I think mainly providing information about administration, routes of administration and how someone can easily swallow. If they do or don’t administer the scheduled medication, or if they leave it with the patient and say ‘just take it later’.”* (Medical specialist 11)

Nurses themselves mentioned how they support pharmacological care by providing and ordering medication; they propose changes of administration routes and support the patient in self-administration of ‘as needed’ medication.

*“Well, we provide information, give explanation about medication use. Yes, we try to assist people to find their way, especially in the use of rescue opioids … some people find that quite difficult, so we intend to guide them. To explain it in such a way that they know when to take a rescue, and provide information to try to eliminate their fear of opioid use or at least reduce it. So mainly in providing information and guide them in using medication.” (*Nurse 2)

Some nurses reported how they intervene in care using non-pharmacological interventions and complementary care.

*“Well sometimes [manage a symptom] with medication, but sometimes with other things, with complementary care. If someone expresses nausea or shows agitation that can be of use.”* (Nurse 7)

### Nurse-informal caregiver

According to all participants, nurses seem to have three roles towards the informal caregiver: to inform, support and involve. Firstly, participants reported a role of the nurse in giving information to the informal caregiver.

*“You can discuss this [discontinuation of medication] with them [informal caregivers] and the nurses can also do that. They are educated well enough to communicate this. And if they [informal caregivers] have questions, we can have a conversation.”* (Medical specialist 9)

Participants reported that nurses inform the informal caregivers especially in case of a patient with reduced consciousness.

*“Well, yes, you do involve them [informal caregivers], mainly inform them, but actually what matters is what the patient wants. But if the patient cannot express himself well, the family serves as spokesperson for the patient. So you try to get clear why people do or don’t want something.”* (Nurse 7)

Secondly, participants reported that nurses support informal caregivers by evaluating informal caregiver capacity and assessing which role informal caregivers can have in medication management. In some cases there was a limited or no role for the nurse because informal caregiver(s) substitute for the nurse.

*“We agreed that the nurses would watch over the medication, because I don’t know anything about that. So if the nurses think medication should be changed they contact the general practitioner.” (*Informal caregiver 18)

Thirdly, in case the patient cannot express his/her wishes or complaints, nurses play a role in involving the informal caregiver and act as a link between the informal caregiver and physician.

*“Well, his daughter-in-law was involved. But she didn’t really interfere in the pills. Her father-in-law could perfectly express himself, so it was not necessary for her to step in at that moment. ( … ) In the course of the admission [the patient’s consciousness reduced and] the nursing staff or I did communicate all medication changes with the family. I discussed major adjustments with the daughter-in-law every time. But about small changes, I think, as a physician I don’t necessarily need to call the family. Then I ask the nurses: “Hey, if you happen to see the daughter-in-law or son, just tell them … ” or “if you meet them, we are satisfied with the situation, are they too?”, because you want their response.”* (Medical specialist 10)

### Nurse-physician

Concerning the relation between physicians and nurses three topics came forward in the responses of the participants: to inform, support and represent. Primarily, nurses monitor the patient and inform the physician about the clinical situation and all aspects related to medication. Moreover, most participants stated that nurses support and complement the physician. Several participants reported how nurses’ informing role results in adaptations in medication management with the aim of better symptom control or reducing polypharmacy.

Interviewer: *“Do you think the physician can also manage the medication without the nurses?”* Patient: *“Yes, but I think he should have way more contact with the patient daily. The nurses observe medication use during the whole week, the effects, how the patient is doing, whether I lie in bed often or not. The physician visits his patient on Fridays with the reports of the nurses. With this he gets down to work, well if he should do that all himself, he can’t do other things. ( … ) So he should be glad that there are nurses doing this kind of work, that he can rely on home-care.” (*Patient 11)

However, the final decision and responsibility for decisions regarding medication is in the hands of the physician.

*“I think that someone [the nurse] who sees the patient every hour should have some influence over part of the decision-making, about how to proceed with that patient. So also about medication use. I think that in the hospital they work as a team. A physician can’t be with the patient constantly, perhaps he sees the patient once a day, but the nurse can. The mandate to make decisions, should be with the physician. Though, in consultation with [the nurse].”* (Informal caregiver 2)

Two aspects regarding the role of the nurse only came forward in the perspective of the physicians. Some physicians think that nurses have a limited role in medication management and reduction of PIMs.

Interviewer: *“And does the nurse play a role in this all?”* Physician: *“In the end it’s all quite complex, even for us physicians it’s difficult to estimate the prognosis, what is useful or not, so I don’t think so. I do think the nurse could discuss this with the patient, whether or not after a first conversation with the physician. So it’s not that I don’t see a role, but not a primary role. I think it’s not up to the nurses to critically review the medication and talk with the patient on their own, because I think they can’t take stock over the situation sufficiently. You can’t demand that from a nurse, I think. So additional explanation in a follow-up conversation fine, that can certainly add value, but not in the decision-making primarily about starting or continuing medication.”* (Medical specialist 2)

In addition, we found physicians who mentioned that the role of the nurses largely depends on their level of education.

*“It depends on who comes, because now and then you see someone involved with lay thoughts about medication. And that doesn’t work. But indeed, in case of a well-educated nurse, she can play a role, for sure. I don’t think that’s a problem at all. Often you know this about people, for whom half a word is enough to get clear if it works or not. But sometimes I see in those home care report folders whole stories about this or that, and that they completely miss the insight into how a drug works.” (*Family physician 1)

Participants expressed that nurses may represent the physician by repeating and clarifying information about medication to the patient. Patients, informal caregivers, nurses and physicians all stressed the importance of frequent and prolonged contact with a health care professional for adequate medication management, and then mainly referred to the nurse. The participants mentioned the daily contact and 24-h availability of nurses as the main factors promoting adequate medication management.

We did not find important differences in the role of the nurses in different settings. However, physicians of patients in hospitals and hospices mostly underlined the value of continuity in nursing care, where physicians of patients residing at home face more challenges in collaborating with different home care teams or organizations.

*“It takes a lot of time to good keep contact. And you should invest a lot to keep it well. Nowadays we have contact with one home care team and that works very well. They give feedback and see things sharply. But to assess for each home care team who you should contact and who not, especially if they have ten different nurses dropping in a week. Yeah, that’s simply undoable, I wouldn’t know how to manage.”* (Medical specialist 17)

## Discussion

In this interview study we assessed the perspectives of patients, informal caregivers, nurses and physicians on the role of nurses in medication management in patients in the last phase of their lives. Based on our results we hypothesize that nurses can have a pivotal role in communication and collaboration regarding medication, with patients, informal caregivers and physicians, by informing, supporting, representing and involving the other parties. Moreover, nurses facilitate adequate pharmacological treatment as part of their regular professional activities and especially with their practical approach.

Sekse et al. in their systematic review stress the unique position of the nurse in palliative care, who holds everything together like a spider in a web, but did not report any involvement of the nurse in medication management [19]. Our findings however suggest that nurses play an important role in symptom management and contribute to improvement of patients’ quality of life at the end of life. Our findings are consistent with the five main principles of symptom management (mnemonic EEMMA): evaluation, explanation, management, monitoring, attention to detail [18]. Specific aspects described by Reid and McCormack could also be identified in our data, for example: a role in evaluating the effectiveness of interventions, in providing information that is vital in decision-making, in management guided by patients’ priorities and goals, in care provision by a cohesive team and in paying attention to detail in their assessment [18]. Others also specifically stress the role of the nurse in being a supporter to the patient [19]. Nurses are considered to have an important role being the ‘eyes and ears’ of the physician and the ‘spokesperson’ for the patient. Physicians may lack attention for the symptom burden in conversations with their patients, because they have to address many other issues. Nurses spend more time with patients and are thus more accessible for patients to discuss their symptoms [19]. In addition, ‘as needed’ or anticipatory medications are often essential to effectively treat symptoms. As nurses are more available, they have a leading role in the administration of anticipatory medications at the end of life [25].

Nurses have a unique opportunity to support adequate medication management and reduction of PIMS as they see patients on a regular basis. They aid in identifying potential effects of changes in the medication regimen on symptoms over time and recognize the shift in goals of care to a palliative approach [20, 22]. As nurses are involved in the administration and/or intake of medication, they directly observe the potential burden of polypharmacy. Nurses can observe difficulties in swallowing medication and inform the physician about the need to adapt the medication or route of administration.

In our study we were not able to account for the level of involvement of the informal caregiver. We did signal an interaction between the involvement of informal caregivers and the nurses, and consequently an influence on how the nurses inform, support and involve the informal caregiver. For informal caregivers palliative care at home is more complex than care in a hospice or a hospital [31, 32]. Tjia at al. describe similar conclusions about the relation between nurses and caregivers at home, based on medication discussions at home visits. They identified those in several domains: teamwork skills, organizational skills, symptom knowledge skills, medication knowledge skills and personhood skills. Thereby they indicate more clearly the complexity of medication management for informal caregivers at home, and how nurses can support in them [31]. In the current literature we notice the explicit role of the nurse in this field, more than involvement of the physicians [19, 32].

Although the final decision about medication (de) prescription is considered the responsibility of the physician, most participants in our study considered medication management a team effort of patient and/or informal caregiver, nurse and physician. This is in line with a study of Riker and Setter on the role of the nurse in medication management in elderly, stating that the physician and the nurse should cooperate to get a complete picture of a patient [20]. The holistic, multidimensional view of the nurse is described as characteristic of their profession [17]. Altogether, the input of the nurse thus supports treatment that is in the patient’s best interest, as described elsewhere for decision-making regarding palliative chemotherapy [23].

However, some physicians interviewed in our study suggested that nurses have a limited or no role in medication management. In a questionnaire study on physicians’ experiences and opinions on medication discontinuation at the end of life, 34% of physicians agreed with the statement ‘nurses’ views about the discontinuation of medications that are not medically necessary are very important to me’ [28]. This might especially hold for situations where nursing staff rapidly turns over, where there is lack of experienced nurses and where the quality of nurses’ assessments varies [21]. In our study some physicians mentioned that the role of the nurse depends on their level of education. This may range from a general nursing training at different levels to specialist palliative care education or an advanced nurse practitioner degree. Knowledge and experience have previously been found to influence the role of the nurse [25], and should be taken into account in further studies. Our results highlight that nurses can have an important role in determining the optimal medication regimen to improve the quality of life of patients at the end of life. Nurses should therefore be involved in medication management. Health care professionals should recognize the role the nurses can have and empower them to contribute to the best possible care for patients at the end of life [19].

### Limitations

Our study involves a secondary analysis of data from interviews on medication management at the end of life; the interviews were not specifically focused on the role of the nurse and therefore we cannot be sure that we achieved saturation on this topic. In our study we only included patients at the end of life with an estimated life expectancy of less than 3 months. Palliative care encompasses the care for all patients with a life-limiting illness, regardless of the life expectancy. The clinical condition of the patient, potential disease-oriented or symptom-oriented treatments, and the expected course of life are only some of the factors that could influence medication management and the role of the nurse. Further, respondents’ statements in the interviews might have been influenced by social desirability. Finally, we were not able to account for differences by care setting or level of education of the nurse in this study, due to the relatively small sample size.

## Conclusions

Medication management in patients with a limited life expectancy involves a complex interplay among patient, informal caregiver, physician and nurse involved. Patients, informal caregivers, physicians and nurses agree that nurses can and should play a role in medication management by informing, supporting, representing and involving the other party. Nurses have a particular role in continuity of care and proximity to the patient, which makes them a valuable intermediary between physician and patient or informal caregiver. Moreover, nurses can contribute to a multidimensional assessment and approach, which is important to promote patients’ interest in medication management at the end of life. Although nurses’ level of involvement may depend on their experience and level of education, physicians should take advantage of their input as physicians themselves may lack awareness of the need of reconsidering the use of medication in patients who are at the end of life. To improve the quality of life of patients at the end of life, nurses should take their role and be reinforced to do so by education and training.

## Data Availability

The datasets generated and analyzed during the current study are not publicly available due to privacy of respondents, but are available from the corresponding author on reasonable request.

## Abbreviations

PIMs: Potentially Inappropriate Medications
MEDILAST: MEDIcation management in the LAST phase of life

## References

[1] Chaudhry SI, Murphy TE, Gahbauer E, Sussman LS, Allore HG, Gill TM (2013). Restricting symptoms in the last year of life: a prospective cohort study. JAMA Intern Med.

[2] Gill TM, Han L, Leo-Summers L, Gahbauer EA, Allore HG (2018). Distressing symptoms, disability, and hospice Services at the end of life: prospective cohort study. J Am Geriatr Soc.

[3] Portz JD, Kutner JS, Blatchford PJ, Ritchie CS (2017). High symptom burden and low functional status in the setting of multimorbidity. J Am Geriatr Soc.

[4] Mercadante S, Casuccio A, Fulfaro F (2000). The course of symptom frequency and intensity in advanced cancer patients followed at home. J Pain Symptom Manag.

[5] Gallagher P, Barry P, O'Mahony D (2007). Inappropriate prescribing in the elderly. J Clin Pharm Ther.

[6] Cruikshank RP, Stafford B, Jones L (2013). Polypharmacy in the terminally ill. Med J Aust.

[7] Todd A, Nazar H, Pearson H, Andrew L, Baker L, Husband A (2014). Inappropriate prescribing in patients accessing specialist palliative day care services. Int J Clin Pharm.

[8] Kotlinska-Lemieszek A, Paulsen O, Kaasa S, Klepstad P (2014). Polypharmacy in patients with advanced cancer and pain: a European cross-sectional study of 2282 patients. J Pain Symptom Manag.

[9] McNeil MJ, Kamal AH, Kutner JS, Ritchie CS, Abernethy AP (2016). The burden of Polypharmacy in patients near the end of life. J Pain Symptom Manag.

[10] LeBlanc TW, McNeil MJ, Kamal AH, Currow DC, Abernethy AP (2015). Polypharmacy in patients with advanced cancer and the role of medication discontinuation. Lancet Oncol.

[11] Arevalo JJ, Geijteman ECT, Huisman BAA, Dees MK, Zuurmond WWA, van Zuylen L (2018). Medication use in the last days of life in hospital, hospice, and home settings in the Netherlands. J Palliat Med.

[12] General Medical Council (2013). Good practice in prescribing and managing medicines and devices.

[13] Geijteman EC, Dees MK, Tempelman MM, Huisman BA, Arevalo JJ, Perez RS (2016). Understanding the continuation of potentially inappropriate medications at the end of life: perspectives from individuals and their relatives and physicians. J Am Geriatr Soc.

[14] Anderson K, Stowasser D, Freeman C, Scott I (2014). Prescriber barriers and enablers to minimising potentially inappropriate medications in adults: a systematic review and thematic synthesis. BMJ Open.

[15] Paque K, Vander Stichele R, Elseviers M, Pardon K, Dilles T, Deliens L (2019). Barriers and enablers to deprescribing in people with a life-limiting disease: a systematic review. Palliat Med.

[16] Dees MK, Geijteman ECT, Dekkers WJM, Huisman BAA, Perez R, van Zuylen L (2018). Perspectives of patients, close relatives, nurses, and physicians on end-of-life medication management. Palliat Support Care.

[17] Witt Sherman D, Free DC, Cherny NI, Fallon M, Kaasa S, Portenoy RK, Currow DC (2015). Nursing and palliative care. Oxford textbook of Palliative medicine.

[18] Reid N, McCormack P, Lugton J, McIntyre RPD (2005). Symptom management: the process of symptom management. Palliative care: the nursing role.

[19] Sekse RJT, Hunskar I, Ellingsen S (2018). The nurse's role in palliative care: a qualitative meta-synthesis. J Clin Nurs.

[20] Riker GI, Setter SM (2013). Polypharmacy in older adults at home: what it is and what to do about it-implications for home healthcare and hospice, part 2. Home Healthc Nurse.

[21] Lim CJ, Kwong MW, Stuart RL, Buising KL, Friedman ND, Bennett NJ (2014). Antibiotic prescribing practice in residential aged care facilities--health care providers' perspectives. Med J Aust.

[22] Turner JP, Edwards S, Stanners M, Shakib S, Bell JS (2016). What factors are important for deprescribing in Australian long-term care facilities? Perspectives of residents and health professionals. BMJ Open.

[23] Nappa U, Rasmussen BH, Axelsson B, Lindqvist O (2014). Challenging situations when administering palliative chemotherapy - a nursing perspective. Eur J Oncol Nurs.

[24] Bowers B, Redsell SA (2017). A qualitative study of community nurses' decision-making around the anticipatory prescribing of end-of-life medications. J Adv Nurs.

[25] Wilson E, Morbey H, Brown J, Payne S, Seale C, Seymour J (2015). Administering anticipatory medications in end-of-life care: a qualitative study of nursing practice in the community and in nursing homes. Palliat Med.

[26] Mercadante S, Prestia G, Casuccio A (2016). Nurse-based monitoring and management of breakthrough pain in an acute pain relief and palliative care unit. Hospital Pract.

[27] Georges JJ, Grypdonck M (2002). Dierckx de Casterle B. being a palliative care nurse in an academic hospital: a qualitative study about nurses' perceptions of palliative care nursing. J Clin Nurs.

[28] Geijteman ECT, Huisman BAA, Dees MK, Perez R, van der Rijt CCD, van Zuylen L (2018). Medication discontinuation at the end of life: a questionnaire study on Physicians' experiences and opinions. J Palliat Med.

[29] Dees MK, Geijteman ECT, Arevalo JJ (2014). Medication management: the opinion of ten experts on medication at the end of life. Pallium..

[30] Rieger KL (2019). Discriminating among grounded theory approaches. Nurs Inq.

[31] Tjia J, Ellington L, Clayton MF, Lemay C, Reblin M (2015). Managing medications during home hospice Cancer care: the needs of family caregivers. J Pain Symptom Manag.

[32] Joyce BT, Lau DT (2013). Hospice experiences and approaches to support and assess family caregivers in managing medications for home hospice patients: a providers survey. Palliat Med.

